# Cracking the Polyketide Code

**DOI:** 10.1371/journal.pbio.0020035

**Published:** 2004-02-17

**Authors:** David A Hopwood

## Abstract

Polyketides, natural products from microorganisms, have been a main source of antibiotics. Understanding the 'programming' of the enzymes that produce these complex molecules has opened a new field of drug discovery

For half a century, natural products from microorganisms have been the main source of medicines for treating infectious disease. The most important chemical class of these antibiotics, apart from the penicillins, is the polyketides. They are made by the stepwise building of long carbon chains, two atoms at a time, by multifunctional enzymes that determine the chain length, oxidation state, and pattern of branching, cyclisation, and stereochemistry of the molecules in a combinatorial fashion to produce an enormous variety of structures. Recent elucidation of the genetic ‘programming’ of the enzymes has opened a new field of drug discovery based on rationally engineering the enzymes to produce ‘unnatural natural products’ with novel properties.

Following the development of penicillin for the treatment of septicemia in the early 1940s, numerous antibiotics were discovered and introduced into medicine. While a fungus makes penicillin, semisynthetic derivatives of which have been a mainstay of antibacterial therapy for decades, most natural antibacterial antibiotics come from a group of soil-dwelling, filamentous bacteria called the actinomycetes, of which Streptomyces is the best-known genus. These organisms make an amazing array of so-called secondary metabolites that have evolved to give their producers a competitive advantage in the complex soil environment, where they are exposed to stresses of all kinds (Challis and Hopwood 2003). The compounds have many functions, but those with antibiotic activity are the most important from the human perspective. Actinomycete antibiotics include such antibacterial compounds as tetracycline and erythromycin, antifungal agents like candicidin and amphotericin, anticancer drugs such as doxorubicin, and the antiparasitic avermectin (Walsh 2003).

While many different chemical classes are represented amongst actinomycete antibiotics, one class accounts for an extraordinary proportion of the important compounds, including all those mentioned above. This chemical family is made up of the polyketides. They are synthesized by multifunctional enzymes called polyketide synthases (PKSs), which are related to the fatty acid synthases that make the lipids essential for the integrity of cell membranes, but they carry out much more complex biosynthetic routines. Repeated rounds of carbon chain building and modification use a series of independently variable reactions selected according to a ‘program’ characteristic of each PKS (Reeves 2003). Recent research has focused on determining this program so as to be able to modify it in rational ways by genetic engineering and thus generate novel drug candidates. The resulting field of ‘combinatorial biosynthesis’ of ‘unnatural natural products’ has been given added urgency by the rise of multidrug-resistant pathogens, of which MRSA (methicillin-resistant Staphylococcus aureus) is simply the most discussed of a series of threats (Walsh 2003). How do PKSs work and how can we make new ones?

## Molecular Diversity

**Figure 1:**
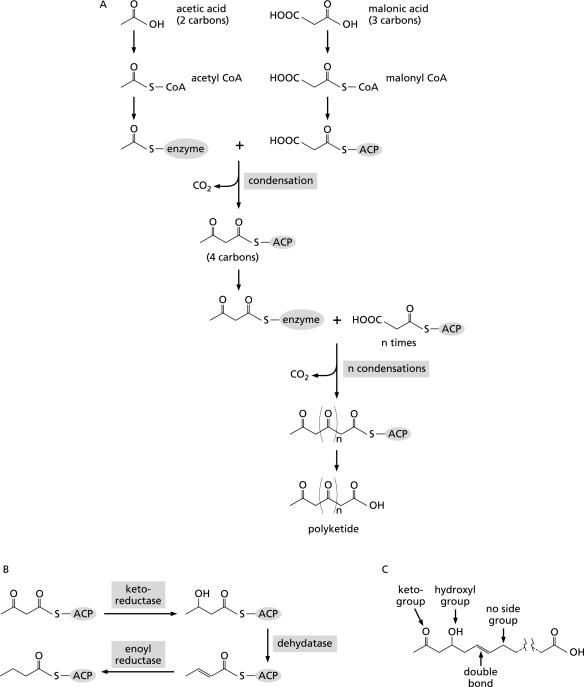
The Chemistry of Polyketide Chain Assembly (A) Acetic acid and malonic acid are converted to their coenzyme A (CoA) esters and then attached, by specific acyl transferases, to components of the polyketide synthase (PKS): acetyl-CoA is attached to the active site of the ketosynthase, and malonyl-CoA to a structural component of the PKS called the acyl carrier protein (ACP). Condensation of the two units by the ketosynthase, with loss of one carbon from malonyl-CoA as carbon dioxide, produces a four-carbon chain attached to the ACP. This is transferred back to the ketosynthase, and further rounds of condensation with malonyl-CoA (as shown) or other chain extender units produce a polyketide chain. (B) The three-step reductive cycle that converts a keto group to a hydroxyl, then to a double bond, and finally to a fully saturated carbon. (C) A complex polyketide in which keto groups, hydroxyl groups, double bonds, and fully saturated carbons occur at different positions along the chain, depending on the operation of the reductive cycle after each condensation.

The heart of PKS function is the synthesis of long chains of carbon atoms by joining (condensing) together small organic acids, such as acetic and malonic acid, by a so-called ketosynthase function. This uses the building units in the form of activated derivatives, called coenzyme A (CoA) esters, so we speak of acetyl-CoA and malonyl-CoA, for example. The special form of condensation that joins them is driven by loss of carbon dioxide. Thus, when acetyl-CoA, with two carbon atoms, joins with malonyl-CoA, with three carbons, one of the latter is lost and a chain of four carbon atoms results (Figure 1A). Further rounds of condensation extend the chain by two carbons at each step. If the chain-extender unit, instead of being malonyl-CoA, is methylmalonyl-CoA, which has four carbon atoms, the linear carbon chain is still extended by two carbons, and the ‘extra’ carbon forms a methyl side branch. More complex extender units produce more complex side branches.

Choices of the number and type of the building units are variables in determining polyketide structure. Another concerns the keto groups (C=O) that appear at every alternate carbon atom in the growing chain as a result of the condensation process (accounting for the name polyketide). They may remain intact. Alternatively, some may be modified or removed by a series of three steps (Figure 1B), any of which may be omitted. This results in keto groups remaining at some points in the chain; hydroxyl groups (–OH), formed by reduction of a keto group, at others; double bonds between some adjacent carbon atoms, resulting from removal of the hydroxyl by loss of water (dehydration); or full saturation with hydrogen atoms elsewhere, arising by ‘enoyl’ reduction of the double bond (Figure 1C). A further variable concerns the stereochemistry of the hydroxyl groups and methyl or other carbon branches, each of which can exist in two possible configurations. Finally, the nascent carbon chain adopts different folding patterns after it leaves the PKS, and ‘tailoring’ enzymes can then add sugars or other chemical groups to it at many alternative positions, enabled by the pattern of chemical reactivity built into the polyketide by the PKS. The challenge has been to understand the programming of the PKS that accounts for this gamut of structural variation.

During the 1990s, the ability to manipulate actinomycete genes, developed over previous decades, mainly using the model species Streptomyces coelicolor (Hopwood 1999), was combined with chemical and biochemical experiments to begin to crack this ‘polyketide code’. The first studies were on organisms making antibiotics of the ‘aromatic’ family, which includes tetracycline and doxorubicin, as well as the model compounds actinorhodin (made by S. coelicolor itself) and tetracenomycin. The main variable in their structure is carbon chain length, with few choices of different building units or keto group modification, so the programming would (in principle) be simple. The DNA sequences responsible for such PKSs revealed sets of genes encoding proteins, including ketosynthases, ketoreductases, and acyl carrier proteins (ACPs) (the unit of the PKS on which the growing carbon chain is tethered; see Figure 1A), that would come together to form a multicomponent PKS resembling a typical bacterial fatty acid synthase. In contrast, the DNA sequence of the gene set for the complex polyketide erythromycin, made by a relative of Streptomyces called Saccharopolyspora erythraea, which has more involved programming, revealed multifunctional proteins with the various enzymic functions carried out by active sites on the same polypeptide chain, as in a mammalian fatty acid synthase.

The big surprise, though, was the finding of six sets, or modules, of such active sites, corresponding to the six rounds of condensation needed to build the carbon chain (Cortes et al. 1990; Donadio et al. 1991). The modules each contain an acyl transferase (to load the extender unit onto the enzyme), as well as a ketosynthase and an ACP domain, together with exactly those reductive activities needed to generate the required pattern of modification of the chain at each step of elongation. Thus was born an ‘assembly line’ model in which the program for the PKS is hardwired into the DNA and expressed in a linear array of active sites (domains) along the giant protein. This consists of the six chain-building modules, preceded by a short module for loading the starter unit and ending in a domain for releasing the completed carbon chain from the PKS. The carbon chain of the polyketide would be assembled and modified progressively as the molecule moved along the protein, interacting with each domain in turn, which would select extender units, make carbon–carbon bonds, and modify keto groups as appropriate, depending on the presence or absence of domains for the three steps in the reductive cycle.

The model arose from the gene sequence, but was rapidly tested by mutating individual domains or adding or deleting whole modules and by observing predicted changes in the polyketide product. Soon, dozens of engineered compounds had been made, and the field mushroomed with the isolation of more and more clusters of genes for complex polyketides that both proved the generality of the model (with minor variations) and filled the need for spare parts for the engineering of countless new polyketides (Shen 2003). Several biotech companies were founded to exploit the potential for drug discovery.

## Aromatic PKS Programming

Meanwhile, the programming of the aromatic PKSs was harder to understand. They had been found to contain only a single ketosynthase, which has to operate a specific number of times to build a carbon chain of the correct length, so how is this determined? How does a single reductive enzyme know which keto groups to modify? And how is the starter unit for building the carbon chain selected (the extender units are normally all malonyl-CoA, so no choice is involved)? Considerable progress had been made in constructing novel compounds by mixing and matching PKS subunits, but this was largely based on empirical knowledge about which components to put together (McDaniel et al. 1995). A specific subunit of the PKS, named the chain length factor (CLF), was deduced to have a major influence on carbon chain length (McDaniel et al. 1993), but this conclusion was not universally accepted in the absence of experimental evidence on its mode of action. Two recent publications by the Khosla laboratory at Stanford University describe significant advances in understanding aromatic PKS programming and promise to turn the spotlight back onto engineered members of this class of compounds as potential drug candidates by allowing rational manipulation of the two key variables: carbon chain length and choice of starter unit.

In the first paper (Tang et al. 2003), the authors explore the hypothesis that the CLF exerts control over carbon chain length by associating closely with the ketosynthase, a protein with which it shares considerable amino acid sequence similarity, giving rise to a channel of a certain size at the interface between the two proteins. By systematically changing amino acids at four key positions in the CLF, the size of the channel was altered. Thus, large amino acid residues in the CLF of a PKS that makes a 16-carbon chain were replaced by less bulky residues found in one that builds a 20-carbon chain, and the chain length of the product increased as expected. The authors propose that the length of the channel is the main factor in controlling the number of chain-extension steps that can take place to fill it. While protein–protein interactions with other PKS subunits may modulate this chain length control, the work represents a major step in understanding and manipulating the chain length of aromatic polyketides.

What about the choice of starter unit? Most aromatic polyketides start with acetyl-CoA. An important earlier publication by Leadlay and colleagues (Bisang et al. 1999) had shown that this is not loaded directly onto the PKS, as had been assumed, but is derived by loss of carbon dioxide from a molecule of malonyl-CoA previously loaded onto the enzyme. This decarboxylation is catalysed by the CLF as an activity independent of its role in influencing carbon chain length. There are, however, certain aromatic polyketides, including the anticancer drug doxorubicin, an antiparasitic agent called frenolicin, and the estrogen receptor agonist R1128, that have different starters.

What Tang et al. (2004) have deduced, as described in this issue of *PloS Biology*, is that the PKSs for these compounds consist of two modules of active sites. The components of each module are not activities carried on the same protein, as in the PKSs for the complex polyketides, but are all separate proteins. They form functional modules nevertheless. The newly recognized modules in the producers of compounds that start with nonacetate units have a dedicated ACP and a special ketosynthase that carries out a first condensation, joining the unusual starter unit to the first malonyl-CoA extender unit. The starter module then hands the resulting ‘diketide’ on to the second module (first reducing it, if appropriate, using reductive enzymes ‘borrowed’ from fatty acid biosynthesis) for typical extension by successive condensation with malonyl-CoA units to complete the chain. If the starter module is not present, the second module defaults to its typical habit of decarboxylating malonyl-CoA to acetyl-CoA and starts the chain with that.

The excitement of the work for biotechnology is that it offers the prospect of engineering promising drug candidates by making novel combinations of starter and extender modules and perhaps of feeding the starter modules with a whole range of unnatural substrates (Kalaitzis et al. 2003). It is encouraging that already, in the proof-of-principle studies reported by Tang et al. (2004), some products with improved in vitro antitumor activity were obtained.

## Abbreviations

ACP: acyl carrier protein
CLF: chain length factor
CoA: coenzyme A
MRSA: methicillin-resistant Staphylococcus aureus
PKS: polyketide synthase
